# Comparative analysis of laparoscopic proximal gastrectomy plus semi-embedded valve anastomosis with laparoscopic total gastrectomy for adenocarcinoma of the esophagogastric junction: a single-center retrospective cohort study

**DOI:** 10.1186/s12957-021-02163-z

**Published:** 2021-02-15

**Authors:** Yupeng Wu, Shihao Zhang, Liting Wang, Xuya Hu, Zhanxue Zhang

**Affiliations:** 1 Gastrointestinal Surgery, The Second Hospital of Hebei Medical University, Shijiazhuang City, Hebei Province China; 2 Urology Surgery, The Second Hospital of Hebei Medical University, Shijiazhuang City, Hebei Province China

**Keywords:** Adenocarcinoma of the esophagogastric junction, Proximal gastrectomy, Semi-embedded valve anastomosis, Reflux esophagitis

## Abstract

**Background:**

We invented a new antireflux anastomosis method for use in proximal gastrectomy for adenocarcinoma of the esophagogastric junction (AEG) and named it semi-embedded valve anastomosis (SEV). This study was conducted to compare and analyze the short-term efficacy and long-term prognosis of this anastomosis reconstruction method versus laparoscopic total gastrectomy (LTG).

**Methods:**

We retrospectively analyzed the general data and surgical outcomes of patients with AEG who underwent three united laparoscopic proximal gastrectomy plus semi-embedded valve anastomosis (TULPG-SEV, *N* = 20) and LTG (*N* = 20) at our hospital from January 2015 to September 2017 and investigated the incidence of postoperative reflux esophagitis and postoperative nutritional status between the two groups. Survival analysis was also performed.

**Results:**

The operative time (178.25 ± 15.41 vs 196.5 ± 21.16 min) and the gastrointestinal reconstruction time (19.3 ± 2.53 vs 34.65 ± 4.88 min) of the TULPG-SEV group were significantly less than that of the LTG group. There was no difference in intraoperative blood loss, length of hospital stay, and postoperative complications. There was no difference in the scores on the postoperative reflux disease questionnaires (RDQs) conducted 1 month (*P* = 0.501), 3 months (*P* = 0.238), and 6 months (*P* = 0.655) after surgery between the TULPG-SEV group and LTG group. Gastroscopy revealed 2 cases of reflux esophagitis (grade B or higher) in each group. The postoperative hemoglobin level was better in the TULPG-SEV group than in the LTG group, and the difference was most noticeable at 1 month after surgery (*P* = 0.024) and 3 months after surgery (*P* = 0.029). The levels of albumin and total protein were not significantly different between the groups. There were more patients with weight loss over 5 kg after surgery in the LTG group than in the TULPG-SEV group (*P* = 0.043). There was no significant difference in the 3-year overall survival rate between the two groups (*P* = 0.356).

**Conclusion:**

SEV has a certain antireflux effect and can reduce the anastomosis time. Proximal gastrectomy may be better than total gastrectomy for maintaining postoperative hemoglobin levels and reducing weight loss.

## Introduction

The esophagogastric junction (EGJ) is the area that connects the distal esophagus and the proximal stomach. In recent years, the incidence of adenocarcinoma of the esophagogastric junction (AEG) has increased. This trend was noted in Asia and Europe [1, 2]. To date, the Siewert (German researcher) classification is the most widely used grading system in the world [3]: Siewert type I refers to adenocarcinoma with the center located 1 to 5 cm above the EGJ, type II refers to adenocarcinoma with the center located between 1 cm above and 2 cm below the EGJ, and type III refers to adenocarcinoma with the center located 2 to 5 cm below the EGJ.

Whether proximal gastrectomy (PG) or total gastrectomy (TG) is more suitable for types II and III AEG remains debatable. To achieve a better radical cure and avoid postoperative reflux esophagitis, most experts agree that laparoscopic total gastrectomy (LTG) [4, 5] with open anastomosis using auxiliary incision anastomosis or complete laparoscopic anastomosis (overlapping and π-shaped anastomoses) is preferred. Although there is controversy, some literature reports indicate that TG reduces the quality of life and nutritional status of patients [6, 7]. Recently, a number of studies have shown that upper gastric cancer with tumor diameter less than 4 cm is associated with an extremely low metastasis rate in stations 5 and 6 lymph nodes [8, 9]. This has become the basis for the feasibility of PG. The fifth edition of the Japanese guideline [10] suggests that PG is feasible for early-stage AEG under the premise of ensuring a negative resection margin. The indications for PG continue to expand, and early AEG is only one indication [11].

Traditional anastomosis between the stomach and the esophageal stump is the most widely used anastomosis method for PG but is associated with a high probability of postoperative gastroesophageal reflux [12]. Many surgeons have explored various types of antireflux procedures to avoid this complication [13–15]. Due to the shortcomings of each method, none of them is widely accepted. By using patented laparoscopic instruments (Chinese patent No. 201220661287.9), we invented a hand-assisted laparoscopic gastrointestinal reconstruction method. The anastomosis method was named semi-embedded valve anastomosis (SEV). Our previous study has shown that this anastomosis has potential anti-reflux effect [16].

The purpose of this study was to explore the feasibility and antireflux effect of our new anastomosis method through a retrospective cohort study. Additionally, we compared the nutritional indicators of patients undergoing three united laparoscopic proximal gastrectomy plus semi-embedded valve anastomosis (TULPG-SEV) and those undergoing LTG.

## Methods

### Patients

From January 2015 to September 2017, a total of 153 patients with Siewert types II and III AEG underwent surgical treatment at the Second Hospital of Hebei Medical University. All procedures were performed by chief surgeons who had same experience level and held the same rank. The exclusion criteria were as follows: patients with Siewert type I AEG; patients with tumor invasion to the surrounding organs and distant metastases; patients with severe respiratory, circulatory, and endocrine diseases; patients undergoing neoadjuvant therapy before surgery; patients undergoing thoracoabdominal surgery; patients undergoing laparotomy; and patients who could not be followed for more than 1 year because their medical documents could not be obtained. According to the above exclusion criteria, patients were excluded from this study for the following reasons: 8 because they were undergoing thoracoabdominal surgery, 16 because they were undergoing laparotomy or conversion to laparotomy, 12 because they were undergoing neoadjuvant treatment, 8 because they had inoperable lesions or extensive metastases were found during surgery, 4 because they were undergoing combined evisceration, and 23 because they could not complete the follow-up. Most patients who underwent laparoscopic PG had early-stage lesions, while patients who underwent LTG had late-stage lesions. Therefore, we conducted 1:1 matching according to preoperative stage, age, and sex to control the baseline balance. Due to the need to study the nutritional indicators of the two groups, we excluded patients who had severe hypoalbuminemia and anemia before surgery and patients who received large amounts of blood product transfusion after surgery. Eventually, 40 eligible patients were enrolled and were divided into two groups: a TULPG-SEV group (20 patients) and a LTG group (20 patients). The staging of gastric cancer was based on the guidelines of the Union for International Cancer Control (eighth edition). This study was approved by the Ethics Committee of the Second Hospital of Hebei Medical University (approval number: 2020-R126).

### Surgical procedure

#### TULPG-SEV

**Fig. 1:**
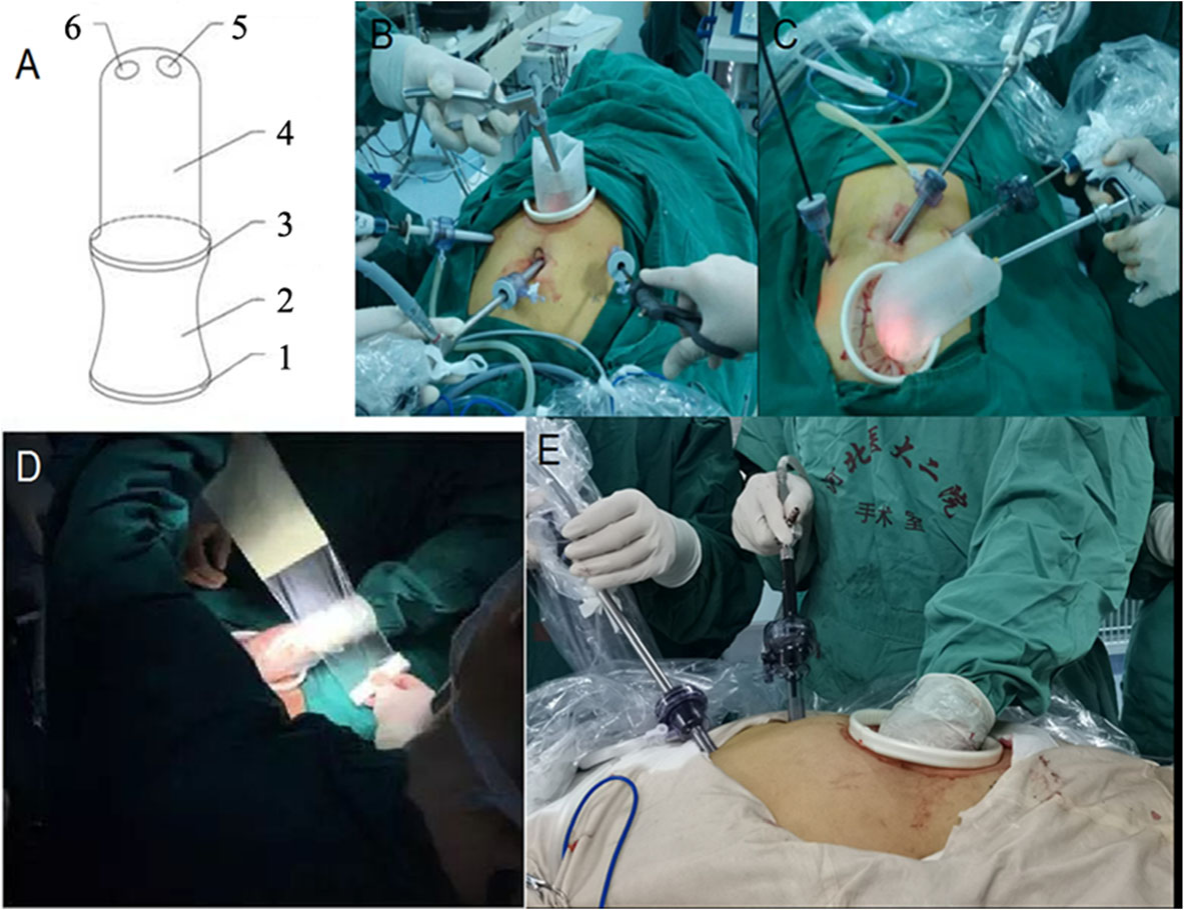
**a** Illustration of the patented device: 1.External elastic ring; 2.Sealed adapter sleeve; 3.Internal elastic ring; 4.Sealing connecting sleeve bag; 5.Cutting mouth for hand; 6.Cutting port for instruments **b**, **c**, **d**, **e** Application of three united laparoscopic surgery to treat adenocarcinoma of the esophagogastric junction

TULPG was developed by the authors and allows the operator to switch freely between single-port laparoscopic surgery, complete laparoscopic surgery, and small-incision hand-assisted laparoscopic surgery during the procedure. The specific method was described in our previous study [16, 17]. In reference to the *Japanese Gastric Cancer Treatment Guideline* (fifth edition), we performed radical PG and completed separation of the stomach, the severing of blood vessels, and the dissection of lymph nodes. Part of the right gastroepiploic arterial arch was preserved on the side with the greater curvature to ensure the blood supply of the remnant stomach. At the time of anastomosis, the abdomen was deflated. A 7-cm incision was made in the middle of the upper abdomen to allow the insertion of the internal elastic ring of the patented hand-assisted device; the external elastic ring was then cut with scissors, the surgeon puts his/her left hand into to the hand-assisted device, and then, the surgical film was used to seal the device. Pneumoperitoneum was re-established. The procedure then switched to hand-assisted laparoscopy (Fig. 1).

##### Key points of SEV [16]

**Fig. 2:**
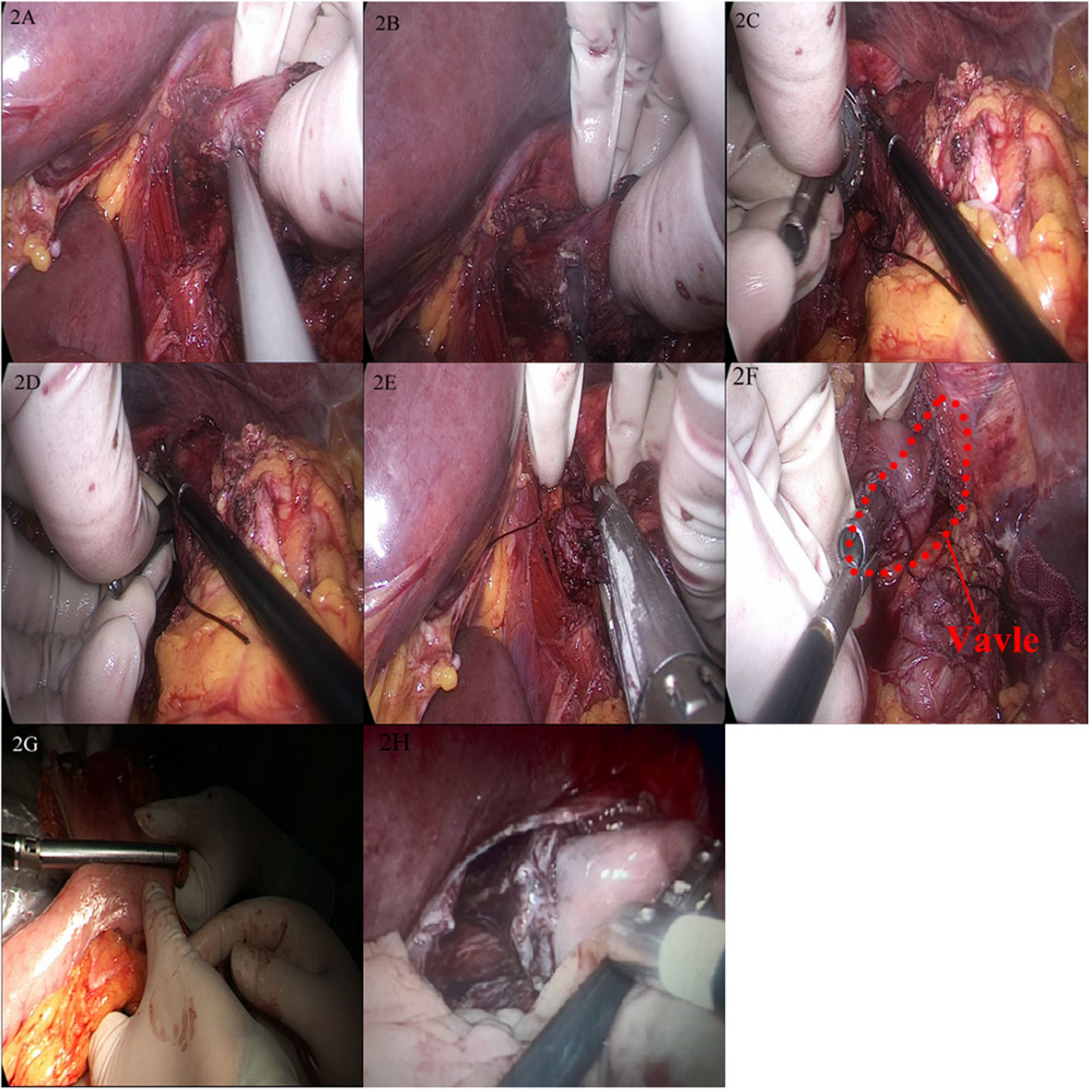
Semi-embedded valve anastomosis surgical procedure. **a**, **b** Hand-assisted incision of the anterior lateral wall of the esophagus. **c**, **d** Placement of the anvil. **e** Forty-five degree cutting with a linear cutting stapler. **e** Reverse puncture using the anvil. **g** Creation of the gastric remnant. **h** Appearance of anastomotic stoma

1. The surgeon localized the upper edge of the tumor with his/her left hand. At a site 2–3 cm from the upper edge, an ultrasonic scalpel was used to make a 1-cm incision in the right wall of the lower esophagus (Fig. 2a).
2. The assistant used laparoscopic instruments to grasp the edge of the incision to open it, and the surgeon slowly inserted the anvil, to which a suture was attached, into the incision (Fig. 2b, c)
3. A 60-mm linear cutting stapler was used to obliquely divide the esophagus at an angle of 45° from the horizontal line, and a small (0.5 cm) opening was left at the lower right corner of the esophageal stump as a reverse puncture port (Fig. 2d).
4. The surgeon pulled the suture attached to the anvil with laparoscopic instruments or his/her hand to make a shaft for the anvil out of the esophageal stump (Fig. 2e, f).
5. The hand-assisted device was removed, and the severed stomach was retracted superiorly. According to the position and size of the tumor, a cutting stapler was placed on the greater curvature side at approximately 45° to the horizontal line to divide the stomach. The resected specimen was removed. A circular stapler was connected to the anvil outside the reverse puncture port for anastomosis of the remnant stomach and the esophageal stump.

**Fig. 3:**
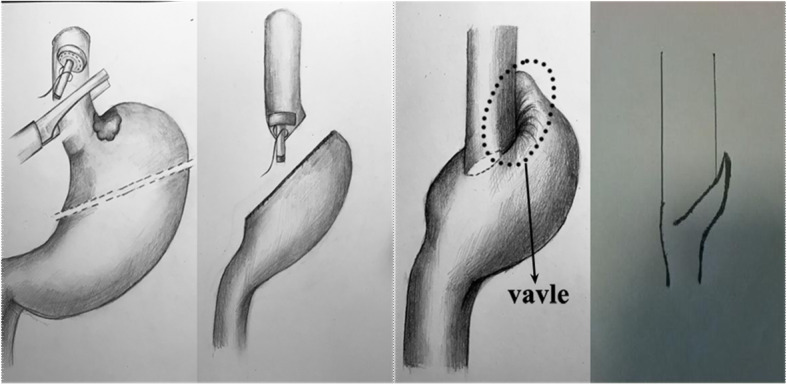
Schematic of the anastomosis and antireflux mechanism

The schematic of the anastomosis is shown in Fig. 3.

#### LTG

According to the requirements of radical TG, we performed standard LTG and D2 lymph node dissection. The lymph node dissection mainly removed the lymph nodes in stations 1, 2, 3, 4sa, 4sb, 4d, 5, 6, 7, 8a, 9, 11p, 11d, and 12a. The left gastric vessel, the right gastric vessel, the left gastroepiploic blood vessel, the right gastroepiploic blood vessel, the short gastric vessel, and the posterior gastric vessel were divided, and the omentum was removed. The jejunum was severed at a site 15–20 cm from the ligament of Treitz. The distal jejunum was anastomosed to the esophagus, and the proximal jejunum was anastomosed to the jejunum approximately 30–40 cm from the esophagojejunostomy site to complete Roux-en-Y reconstruction.

### Data collection and follow-up

#### Surgical and postoperative data

The operative time, intraoperative blood loss, number of lymph nodes harvested, postoperative length of hospital stay, and postoperative complications of the two groups of patients were collected through the electronic medical record system and anesthesia records. The time required for digestive tract reconstruction was recorded during the operation.

#### Nutrition and gastroesophageal reflux indicators

Nutritional indicators (levels of albumin, total protein, and hemoglobin) were documented before surgery. At the sixth month after operation, the weight loss was recorded. The patients were asked to come to the hospital or visit another hospital for follow-up examination 1, 3, 6, and 12 months after surgery. The levels of albumin, total protein, and hemoglobin were recorded, and the reflux disease questionnaire (RDQ) was evaluated. The details of the questionnaire can be found in the literature [18]. Electronic gastroscopy was performed 6 months after the operation, and the Los Angeles grade of gastroesophageal reflux [19] was recorded.

Follow-up was performed for all patients after discharge from the hospital via telephone, outpatient visit, and inpatient examination. In addition to the abovementioned nutritional and gastroesophageal reflux indicators, we also recorded the data of recurrence and death. The follow-up period was at least 3 years.

### Statistical methods

Statistical analysis was performed using the SPSS 22.0 software. Quantitative data with a normal distribution are expressed as mean ± standard deviation (x ± s), and independent samples *t*-tests were used for data comparisons between two groups. Quantitative data with a non-normal distribution are expressed as median (range). The Mann-Whitney *U* test was used for data comparisons between two groups. Qualitative data are expressed as rate. The chi-squared test, chi-squared correction test, or Fisher’s exact test was used to compare differences between groups. The Kaplan-Meier method was used to describe 3-year overall survival with survival curves. The log-rank test was used to compare overall survival differences between the groups. *P* < 0.05 was considered statistically significant.

## Results

### Patient characteristics

A total of 40 patients were included in this study, including 20 patients in the TULPG-SEV group and 20 patients in the LTG group. The general information and baseline characteristics are listed in Table 1. There were no significant differences in age, sex, body mass index (BMI), and pathological type between the groups. In terms of preoperative staging, there were more late-stage patients in the LTG group than in the PG group, although the difference was not significant (*P* = 0.356), and there were no stages 1A and 1B patients in the LTG group. There were more Siewert type III patients in the LTG group than in the PG group, but the difference was not significant (*P* = 0.091). The tumor diameter in the LTG group was slightly larger than that in the TULPG-SEV group (*P* = 0.016).

**Table 1:** Basic characteristics of the patients

Variables	TULPG + SEV (*n* = 20)	LTG (*n* = 20)	*P*-value
Sex			
Male	15	15	1.0
Female	5	5	
Age, mean ± SD [years]	63.2 ± 6.7 (48–75)	61.0 ± 10.9 (32–72)	0.447
BMI, mean ± SD [kg/m^2^]	24.3 ± 1.8	23.8 ± 1.6	0.357
ASA score			
0	12	10	0.748
1	7	8	
2 or more	1	2	
UICC stage			
≤ II A	10 (IA = 2, IB = 3, IIA = 5)	6 (IA = 0, IB = 0, IIA = 6)	0.356
II B	7	8	
III A	3	6	
Pathological type			
Differentiated	5	7	0.630
Undifferentiated	13	10	
Others	2	3	
Tumor size (cm)	3.3 ± 1.2	4.8 ± 1.4	0.016
Siewert type			
II	9	4	0.091
III	11	16	

*TULPG+SEV* three united laparoscopic proximal gastrectomy plus semi-embedded valve anastomosis, *LTG* laparoscopic total gastrectomy, *BMI* body mass index, *ASA* American Society of Anesthesiologists, *UICC* Union for International Cancer Control

### Surgical outcomes and postoperative complications

All 40 patients successfully underwent surgery without conversion to laparotomy. The surgical outcomes and postoperative complications are shown in Table 2. The operative time (178.25 ± 15.41 vs 196.5 ± 21.16 min, *P* = 0.03) and the time for digestive tract reconstruction (19.3 ± 2.53 vs 34.65 ± 4.88 min, *P* < 0.01) in the TULPG-SEV group were significantly shorter than those in the LTG group. The number of lymph nodes harvested was greater in the LTG group than that in the TULPG-SEV group (24.65 ± 2.96 vs 20.45 ± 2.42, *P* < 0.01) because lymph node dissection (stations 5 and 6) was routinely performed in the LTG group. The remaining parameters, including intraoperative blood loss (*P* = 0.640) and postoperative length of hospital stay (*P* = 0.947), were not significantly different between the groups. A positive surgical margin was reported in a patient with Siewert type II AEG. There was no significant difference in early postoperative complications between the two groups (*P* = 0.658). A patient in the TULPG-SEV group had postoperative gastroparesis and was treated with fasting, parenteral nutrition, gastrointestinal decompression, gastrointestinal stimulants, and traditional Chinese medicine acupuncture. This patient was discharged 40 days after treatment. Postoperative pneumonia was reported in one patient. Anastomotic leakage was reported in 2 patients in the LTG group. The 2 patients were discharged 20 and 45 days after conservative treatment. Incision infection was reported in 2 patients. In terms of long-term postoperative complications, there was no significant difference between the two groups (*P* = 0.405). There was one case of anastomotic stenosis in the TULPG-SEV group that was cured after endoscopic balloon dilatation. Anastomotic ulcers were identified under electron microscopy in 4 and 3 patients in the LTG and TULPG-SEV group, respectively.

**Table 2:** Surgical outcomes and complications of the patients

	TULPG-SEV (*n* = 20)	LTG (*n* = 20)	*P* value
Surgical outcomes			
Operative time (minutes)	178.25 ± 15.41	196.5 ± 21.16	0.03
Blood loss (ml)	100 (50–400)	105 (50–800)	0.640
Number of harvested lymph nodes (*n*)	20.45 ± 2.42	24.65 ± 2.96	< 0.01
Postoperative hospital stay (days)	12.5 (9–40)	12 (8–45)	0.947
Time for digestive tract reconstruction	19.3 ± 2.53	34.65 ± 4.88	< 0.01
Early postoperative complications	2	4	0.658
Anastomotic leakage	0	1	
Anastomotic bleeding	0	0	
Abdominal abscess	0	0	
Pancreatic fistula	0	0	
Intra-abdominal infection	0	1	
Small bowel obstruction	0	0	
Pneumonia	1	0	
Surgical site infection	0	2	
Delayed gastric emptying	1	0	
Late postoperative complications	5	3	0.405
Anastomotic stricture	1	0	
Anastomotic ulcer	4	3	
Internal hernia	0	0	
Cholecystitis	0	0	

*TULPG+SEV* three united laparoscopic proximal gastrectomy plus semi-embedded valve anastomosis, *LTG* laparoscopic total gastrectomy

### Indicators related to gastroesophageal reflux

The RDQ questionnaire scores and electronic gastroscopy findings of reflux disease at different times after surgery in each patient are shown in Table 3. The RDQ scores 1 month after surgery (6.5 ± 4.75 vs 5.65 ± 2.96, *P* = 0.501), 3 months after surgery (6 ± 2.63 vs 7.05 ± 2.89, *P* = 0.238), and 6 months after surgery (5.25 ± 2.14 vs 4.95 ± 2.06, *P* = 0.655) were not significantly different between the TULPG-SEV and LTG groups. One patient in the TULPG-SEV group had a score of 20 1 month after surgery, and obvious symptoms of gastroesophageal reflux were present. The patient received oral proton pump inhibitor (PPI) regularly. The symptoms were well controlled, and the score dropped to 12 at 6 months after surgery. It is worth noting that there were two patients in the LTG group with scores of 15 and 10, respectively, which are considered indicative of alkaline reflux esophagitis. The trend reflected in both groups was that the scores of patients with reflux symptoms decreased over time and with regular medication use.

**Table 3:** Postoperative reflux disease (RDQ) questionnaire scores and electronic gastroscopy findings

	TULPG + SEV (*n* = 20)	LTG (*n* = 20)	*P* value
RQD score			
1 month after operation	6.5 ± 4.75	5.65 ± 2.96	0.501
3 months after operation	6 ± 2.63	7.05 ± 2.89	0.238
6 months after operation	5.25 ± 2.14	4.95 ± 2.06	0.655
Postoperative endoscopic findings^a^	2	2	1.000
B	1	2	
C or D	1	0	

*TULPG+SEV* three united laparoscopic proximal gastrectomy plus semi-embedded valve anastomosis, *LTG* laparoscopic total gastrectomy, *RDQ* reflux diagnostic questionnaire

^a^Los Angeles classification

Forty patients underwent electronic gastroscopy 6 months after surgery. According to the Los Angeles gastroesophageal reflux classification, there was one patient with grade B reflux and one patient with grade C reflux in the TULPG-SEV group, while there were 2 patients with grade B reflux in the LTG group. Regarding the incidence of gastroesophageal reflux worse than grade B, the two groups were not significantly different (*P* = 1.000).

### Nutrition-related indicators

**Fig. 4:**
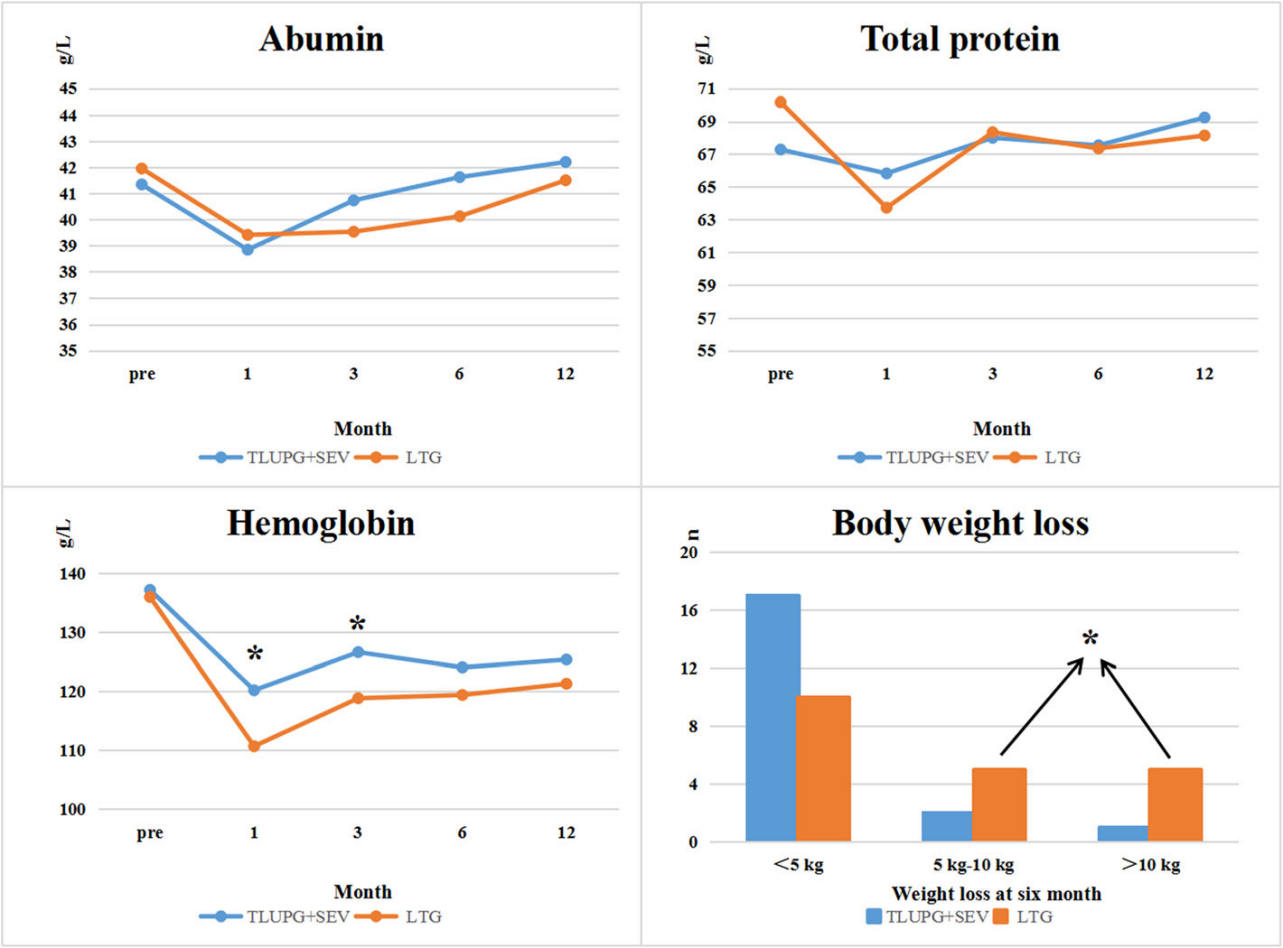
Nutritional indicators after TULPG-SEV and LTG (albumin, hemoglobin, total protein, weight loss). TULPG+SEV, three united laparoscopic proximal gastrectomy plus semi-embedded valve anastomosis; LTG, laparoscopic total gastrectomy. *P less than 0.05

The nutritional indicators of the two groups are shown in Fig. 4. Compared with the preoperative hemoglobin level, each group showed a significant decrease trend after the operation, with a more noticeable decrease in the LTG group than in the TULPG-SEV group (1 month after surgery 120.15 vs 110.65 g/L, *P* = 0.024; 3 months after surgery: *P* = 0.029). In the LTG group, two patients needed oral anti-anemia drugs. One year after surgery, hemoglobin level was still better in the TULPG-SEV group than in the LTG group, but this difference was not significant (125.3 vs 121.25 g/L; *P* = 0.081). The albumin level of the two groups also showed a downward trend at 1 month after surgery and gradually returned to the preoperative level over time. However, TULPG-SEV did not show any advantage compared with LTG at any time point in terms of albumin level or total protein level. The number of patients with weight loss over 5 kg at the sixth month after operation in the TULPG-SEV group was significantly smaller than that in the LTG group (3 vs 10, *P* = 0.043).

### Follow-up

**Fig. 5:**
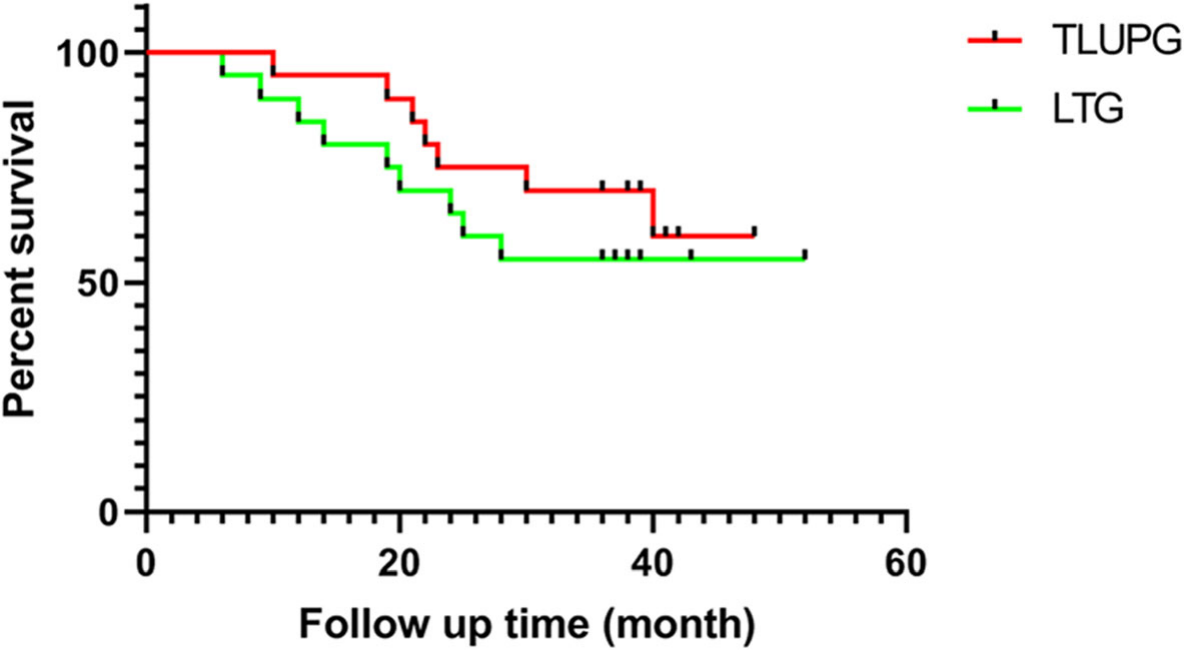
Survival curve after TULPG-SEV and LTG. TULPG, three united laparoscopic proximal gastrectomy; LTG, laparoscopic total gastrectomy

All 40 patients successfully completed follow-up by telephone survey and outpatient visit. No patients were lost to follow-up. The effective follow-up rate was 100%. The median follow-up period was 36 months (range 6–52). There was no significant difference in 3-year overall survival between the two groups (65% vs 55%, *P* = 0.356). The survival analysis is shown in Fig. 5.

## Discussion

Because of its special position and unique biological behavior, AEG is different from lower esophageal and gastric adenocarcinoma. It needs to be treated as an isolated disease [20]. Comprehensive treatment based on radical surgery is an effective method for treating AEG. With the development of laparoscopic surgery and the improvement of surgical techniques, the advantages of laparoscopic surgery in the treatment of early and advanced gastric cancer have been confirmed, for instance, in the KLASS-02 study in South Korea and the JLSSG0901 study in Japan [21, 22]. The lymph node metastasis pattern of types II and III AEG is similar to that of gastric adenocarcinoma. The tumor mainly metastasizes to the abdominal cavity and lower mediastinum and is surgically treated by general surgeons via a transesophageal hiatus (TH) approach or thoracoabdominal surgery.

The main surgical methods for nonadvanced patients with Siewert types II and III AEG include PG and TG. In the past, experts have advocated that TG has advantages in terms of sufficient resection, more thorough lymph node dissection, and better radical cure; however, the in-depth study of Siewert types II and III AEG lymph node metastasis has proven that this stance is incorrect. A multicenter prospective study in Japan [23] showed that AEG (CT2-T4, average tumor diameter 4.6 cm) was associated with very low rate of lymphatic metastasis to stations 5 (1.1%) and 6 (1.7%). Another study that included 2807 cases also reported similar conclusions [24]. Our study demonstrated that there was no significant difference in the 3-year overall survival between the PG and TG groups (65% vs 55%, *P* = 0.356), and this finding is consistent with the results of a meta-analysis by Chen et al. [25].

### PG and reflux esophagitis

Esophagogastrostomy (EG) is the simplest and most widely used method for digestive tract reconstruction after PG. However, traditional EG is associated with a high probability of intolerable gastroesophageal reflux after surgery. A number of studies have shown that the probability is approximately 22–71% [12, 26–28]. The causes of gastroesophageal reflux are as follows: (1) During PG, the lower esophageal sphincter is removed, and thus, the angle of His is destroyed, and the physiological antireflux function of the cardia is lost. (2) The residual stomach still secretes gastric acid, but the reduction of the gastric cavity causes slow emptying. (3) The division of the vagus nerve weakens gastric motility and causes persistent contraction of the pylorus, which further increases the probability of gastroesophageal reflux disease.

The anastomosis method described in the present study is inspired by the functioning of a heart valve, which can only open in one direction and thus can prevent the back flow of blood. We create a valve-like structure in the junction between the esophagus and the gastric remnant to prevent the reflux of gastric content.

The mechanism of this antireflux procedure is as follows: (1) The increase in pressure in the remnant stomach exceeds the pressure in the esophagus to create a pressure difference. When the pressure difference increases, the valve-like structure gradually closes, thus playing a role in preventing reflux. (2) When abdominal pressure increases, the muscle tension at the diaphragmatic hiatus will increase and act on the valve-like structure at the esophageal hiatus to close and prevent reflux.

### Comparison with other antireflux methods

The Japanese researchers Kamikawa et al. [29] first reported a double-flap antireflux method in 2001 that is also known as Kamikawa anastomosis. Muraoka et al. [30] documented 17 cases of laparoscopic double-flap anastomosis and found only one case of grade B reflux esophagitis and one case of minor anastomotic leakage. Moreover, they observed the postoperative formation of an “artificial cardia” via endoscopy. However, their average operative time was 372.4 min. In contrast, in our study, the operative time of the TULPG-SEV group was only 178.25 ± 15.41 min. The shorter operative time indicates a shorter intraoperative anesthesia time, which can reduce the intraoperative risk. Yamashita et al. [31] reported for the first time in 2016 an antireflux procedure called “side-overlap” that was performed under full laparoscopic surgery. The essence of this procedure is a folded anastomosis of the lateral gastroesophageal wall. Their results showed that only one out of 14 patients in the group that underwent this procedure had reflux esophagitis. It is the author’s understanding that the advantages of the side-overlap anastomosis method are as follows: (1) It is a full laparoscopic procedure, which is less traumatic to the patient and is conducive to postoperative recovery; (2) a linear cutting stapler is used for anastomosis because it creates a large anastomosis; anastomotic stenosis may not occur. The disadvantage lies in the need to preserve more than a 5-cm abdominal segment of the esophagus, which makes it suitable only for Siewert type III AEGs with a low tumor position. Our SEV is also suitable for some high-position Siewert type II AEGs because the final anastomosis uses a circular stapler, which can perform high-position cutting.

Jejunum interpositioning and double-tract reconstruction are similar methods [7, 13]. Their antireflux approach involves inserting a pedicled jejunum between the stomach remnant and the esophagus, and using the length gradient of the inserted jejunum and the alkaline liquid of the intestinal fluid to neutralize gastric acid to create an antireflux effect. However, these two anastomosis reconstruction methods eventually create three anastomoses, which may increase the incidence of anastomotic-related complications. In our study, no anastomotic leakage was reported in the 20 patients in the TULPG group, but anastomotic stenosis occurred in one patient.

### TULS (three united laparoscopic surgery)

Minimally invasive surgeries for gastric cancer include laparoscopic-assisted, hand-assisted laparoscopic, complete laparoscopic, single-port laparoscopic radical gastric cancer, and even robotic surgery. Each minimally invasive method has its optimal indications and limitations. Da Vinci robotic surgery requires robotic equipment and has not been popularized worldwide. Our patented device can switch freely between single-port laparoscopy, complete laparoscopy, and small-incision hand-assisted laparoscopy, and we named it TULS [16, 17]. It is especially suitable for procedures involving in complicated abdominal conditions and combined evisceration. The advantages of converting to hand-assisted laparoscopy during anastomosis are as follows: (1) It is very convenient to place the stapler anvil and perform reverse puncture by hand. This can shorten the operative time. (2) It is convenient to assess the length of the esophagus with tumor invasion by hand; furthermore, doing so can improve the R0 resection rate of the upper surgical margin.

### Nutrition after proximal gastrectomy

In the field of tumor treatment, while radical treatment and long survival after surgery are the main goals, increasing attention is being paid to improving the quality of life of patients after surgery. The advantages of proximal gastrectomy are as follows: (1) It preserves the distal stomach that retains some function for food storage, preliminary digestion, and absorption; (2) It preserves the original physiological pathway of food through the duodenum and has less impact on gastrointestinal neuroendocrine and gastrointestinal hormone peptides; 3. It preserves some of the parietal cells, which can secrete intrinsic factors that can help to absorb vitamin B12 and reduce the probability of postoperative megaloblastic anemia. The study by Masuzawa et al. [32] demonstrated that proximal gastrectomy is superior to total gastrectomy in terms of serum indicators (hemoglobin, albumin). The study by Nishigori et al. [33] showed that postoperative weight loss and gastrointestinal symptoms were significantly reduced with proximal gastrectomy compared to total gastrectomy. Our study has also confirmed that TULPG is related to high levels of hemoglobin and reduce weight loss compared to LPG. The number of patients with postoperative weight loss greater than 5 kg at the sixth month after operation in TULPG group was significantly less than that in LTG group (3 vs 10, *P* = 0.043). However, TULPG was not superior to LPG in albumin and total protein indicators. This result is different from the abovementioned results. This may be due to the smaller number of cases we included. In addition, a few studies [34, 35] show that PG and TG have no significant differences in terms of changes in nutritional indicators.

### Limitations of this study

First, as of 2020, the number of SEV cases that we have completed is limited. Thus, this study has a small sample size. In addition, 23 patients who could not be followed up for more than 1 year were excluded, but this would lead to selective bias. Second, regarding the results for nutrition-related indicators, some patients require oral Xelox for chemotherapy, which may affect hemoglobin, albumin, body weight and other indicators. Prospective multicenter studies are still needed to verify the feasibility and antireflux effect of this surgical method.

## Conclusion

SEV is a simple and feasible PG approach for the treatment of AEG. It has a short anastomosis reconstruction time and offers some preventive effect against postoperative reflux esophagitis. PG has better results than TG in terms of maintaining postoperative body weight and reducing anemia.

## Data Availability

The datasets generated and/or analyzed during the current study are not publicly available due to protecting individual patient privacy but are available from the corresponding author on reasonable request.

## Abbreviations

BMI: Body mass index
ASA: American Society of Anesthesiologists
UICC: Union for International Cancer Control
EGJ: Esophagogastric junction
AEG: Adenocarcinoma of the esophagogastric junction
PG: Proximal gastrectomy
TG: Total gastrectomy
LTG: Laparoscopic total gastrectomy
SEV: Semi-embedded valve anastomosis
TULPG: Three united laparoscopic proximal gastrectomy
RDQ: Reflux disease questionnaire
PPI: Proton pump inhibitor
EG: Esophagogastrostomy
TULS: Three united laparoscopic surgery
